# Supplementary material for: Impact of Early Nutrient Intake and First Year Growth on Neurodevelopment of Very Low Birth Weight Newborns
Source: Nutrients. 2022 Sep 6;14(18):3682. doi: 10.3390/nu14183682 (PMC9506449; doi:10.3390/nu14183682)
Supplement: Supplementary file 1 [file nutrients-14-03682-s001.zip › nutrients-1900939-supplementary.pdf]

Supplementary Table S1. Parenteral nutrient and parenteral fluid intake, and human milk intake on days of life 1, 4 and 7. * 12PM to 12PM were used for 24–hour calculations. If the infant was born after 12PM, first day's actual milk intake was calculated as total and not adjusted for 24 hours. Values are median (interquartile range).

Indicator	Extremely Preterm (*n*=41)	Very Preterm (*n*=54)	*p*
Day 1			
Amino acids, g/kg/day	2.8 (2.7–3)	2.8 (2.6–2.9)	0.265
Carbohydrates, g/kg/day	7.1 (6.7–7.9)	7.0 (6.6–7.3)	0.221
Fat, g/kg/day	1.0 (0.9–1.0)	1.0 (0.9–1.0)	0.879
Energy, kcal/kg day	46 (43–48)	44 (43–46)	0.165
Parenteral fluids, mL/kg/day	81 (79–85)	80 (76–82)	0.112
Milk volume day 1, mL/kg/day	8 (5–13)*	11 (7–15)*	0.044
Day 4			
Amino acids, g/kg/day	2.3 (1.9–3.0)	1.6 (1.1–2.0)	<0.001
Carbohydrates, g/kg/day	7.4 (6.4–9.2)	5.4 (3.5–7.7)	<0.001
Fat, g/kg/day	1.9 (1.3–2.6)	1.5 (0.9–2.0)	0.027
Energy, kcal/kg day	54 (41–65)	39 (26–55)	<0.001
Parenteral fluids, mL/kg/day	86 (77–105)	60 (35–74)	<0.001
Milk volume day 4, mL/kg/day	57 (36–69)	77 (55–92)	<0.001
Day 7			
Amino acids, g/kg/day	1.0 (0.0–2.0)	0	<0.001
Carbohydrates, g/kg/day	3.7 (0.0–7.0)	0	<0.001
Fat, g/kg/day	0.2 (0.0–1.3)	0	<0.001
Energy, kcal/kg day	54 (41–65)	0	<0.001
Parenteral fluids, mL/kg/day	34 (0–69)	0	
Milk volume day 7, mL/kg/day	124 (91–140)	144 (127–157)	<0.001

Supplementary Table S2. Total nutrient intake during the first 4 weeks. Values are median (interquartile range). * Week 1 intake are underestimated and represent mainly parenteral intake.

Indicator	Extremely Preterm (*n* = 41)	Very Preterm (*n* = 54)	*p*
Week 1 *			
Total protein, g/kg/day	2.5 (2.2–2.8)	1.9 (1.4–2.4)	<0.001
Total carbohydrates, g/kg/day	8.9 (7.3–10.48)	7.1 (5.3–9.0)	<0.001
Total fat, g/kg/day	2.4 (1.8–3.0)	2.0 (1.3–2.8)	0.170
Total energy, kcal/kg/day	66 (56–78)	56 (38–73)	0.016
Week 2			
Total protein, g/kg/day	3.3 (2.7–3.7)	3.6 (3.2–4.0)	0.011
Total carbohydrates, g/kg/day	12.8(11.8–14.4)	14.2 (12.8–15.5)	0.009
Total fat, g/kg/day	5.8 (5.1–6.7)	5.7 (4.9–6.4)	0.432
Total energy, kcal/kg/day	121 (108–135)	127 (119–139)	0.127
Week 3			
Total protein, g/kg/day	3.6 (3.0–3.8)	3.8 (3.1–4.0)	0.168
Total carbohydrates, g/kg/day	15.4(13.1–16.4)	15.9 (14.5–16.7)	0.115
Total fat, g/kg/day	6.1 (5.6–7.5)	6.4 (5.6–7.4)	0.886
Total energy, kcal/kg/day	131 (119–145)	137 (127–148)	0.174
Week 4			
Total protein, g/kg/day	3.4 (3.1–3.8)	3.4 (2.6–3.8)	0.890
Total carbohydrates, g/kg/day	16.0 (13.8–16.6)	15.9 (14.1–16.7)	0.641
Total fat, g/kg/day	6.2 (5.2–7.7)	6.3 (5.4–6.7)	0.783
Total energy, kcal/kg/day	137 (118–150)	134 (124–14)	0.830

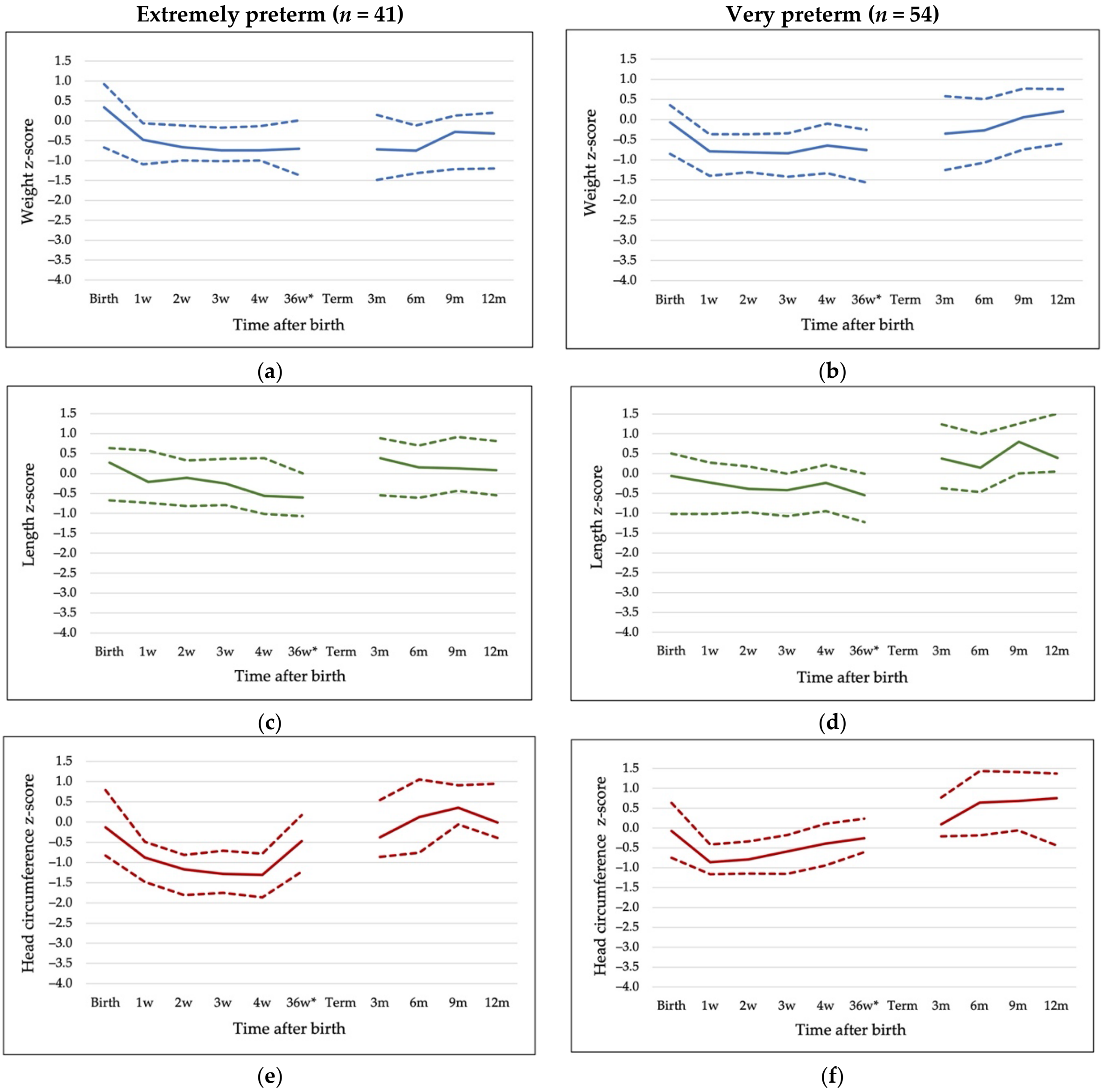

Supplementary Figure S1. Growth patterns of groups during the first year of life. (**a**)—weight z-score in EP group, (**b**)—weight z-score in VP group, (**c**)—length z-score in EP group, (**d**)—length z-score in VP group, (**e**)—head circumference z-score in EP group, (**f**)—head circumference z-score in VP group. Values are median (solid line) and interquartile range (dashed lines). Z-scores from birth to discharge were calculated using Fenton (2013) reference, z-scores 3 to 12 months were calculated using the WHO (2006) reference. 36w*—36 weeks of corrected gestational age or discharge.

Supplementary Table S3. Relationship between growth by z-scores (Fenton 2013) during hospitalization period, average daily total nutrient intake during first 28 days, and neurodevelopment at 12 months of CGA.

**Model 1.**	**MDI**			**PDI**		
	$R = 0.695, R^2 = 0.483$			$R = 0.513, R^2 = 0.263$		
**23–27 weeks**	B	β_s	p	B	β_s	p
Sex	−1.55	−0.07	0.646	−0.39	−0.16	0.930
Gestational age, weeks	1.16	0.01	0.493	0.84	0.07	0.707
Birth weight z-score	−4.26	−0.35	0.040	−0.56	−0.04	0.832
Change of weight z-score birth to discharge	0.885	0.60	0.774	−3.04	−0.19	0.457
Change of length z-score birth to discharge	2.12	0.16	0.377	3.45	0.23	0.277

Change of HC z-score birth to discharge	2.27	0.14	0.377	0.47	0.03	0.888
Total protein, g/kg/day	−5.76	−0.22	0.358	−1.57	−0.05	0.848
Total carbohydrates, g/kg/day	4.26	0.62	0.008	2.37	0.31	0.239
Total fat, g/kg/day	0.88	0.07	0.707	3.03	0.22	0.331
	**MDI**			**PDI**		
	R = 0.362, R^2 = 0.131			R = 0.349, R^2 = 0.122		
**28–34 weeks**	B	β_s	*p*	B	β_s	*p*
Sex	6.02	0.26	0.121	2.99	0.12	0.468
Gestational age, weeks	−1.50	−0.21	0.545	0.00	0.00	0.999
Birth weight z-score	−3.20	−0.26	0.453	1.89	0.14	0.679
Change of weight z-score birth to discharge	−1.76	−0.07	0.725	−1.43	−0.05	0.790
Change of length z-score birth to discharge	3.71	0.17	0.394	3.58	0.15	0.443
Change of HC z-score birth to discharge	2.03	0.11	0.509	0.64	0.03	0.846
Total protein, g/kg/day	−4.57	−0.18	0.457	3.61	0.13	0.584
Total carbohydrates, g/kg/day	2.10	0.26	0.323	1.84	0.21	0.421
Total fat, g/kg/day	−0.90	−0.08	0.672	−3.10	−0.25	0.178
